# Supplementary material for: Mitochondrial Genome Fragmentation Occurred Multiple Times Independently in Bird Lice of the Families Menoponidae and Laemobothriidae
Source: Animals (Basel). 2023 Jun 20;13(12):2046. doi: 10.3390/ani13122046 (PMC10295106; doi:10.3390/ani13122046)
Supplement: Supplementary file 1 [file animals-13-02046-s001.zip › animals-2410596-supplementary.pdf]

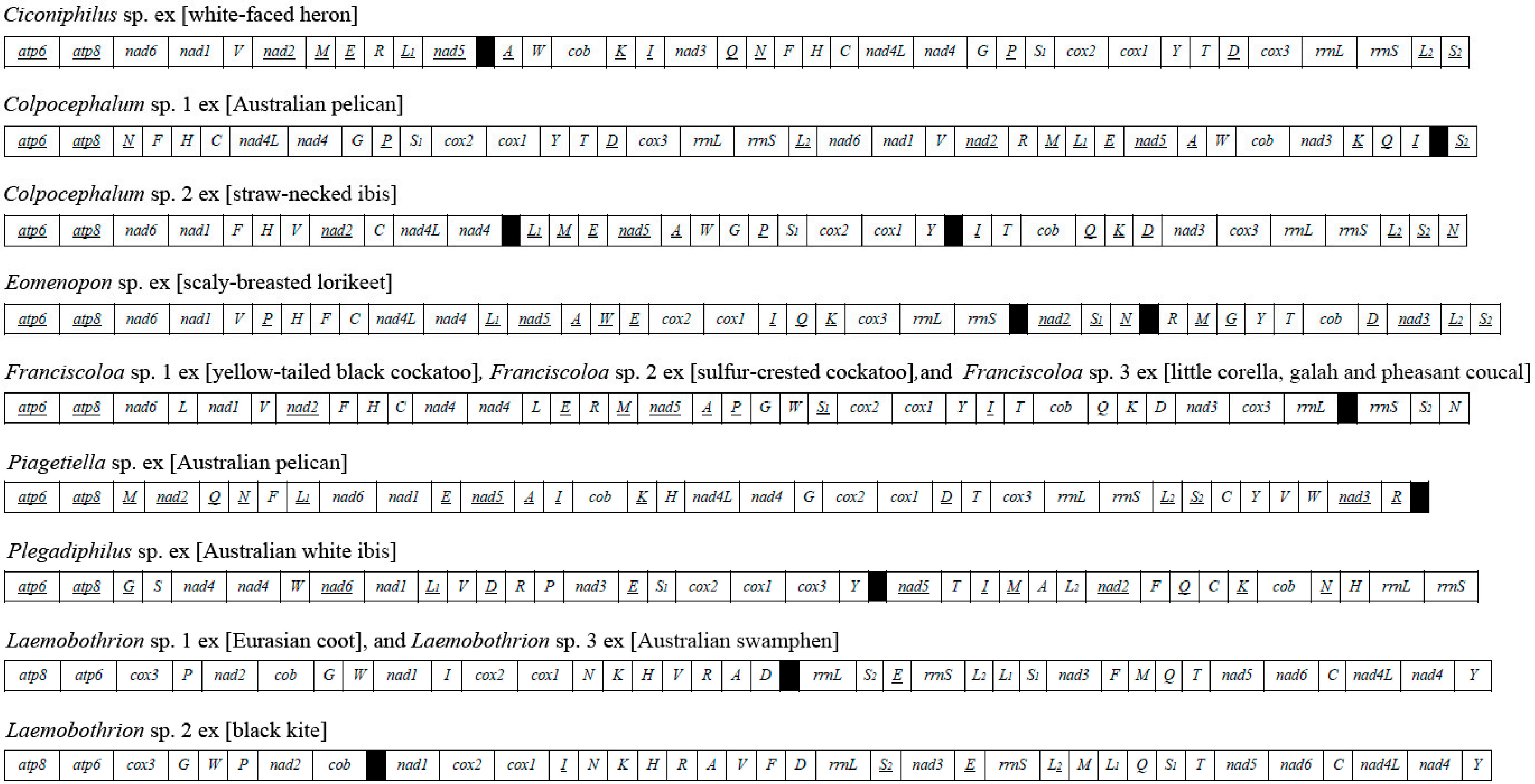

Supplementary Figure S1. The mitochondrial genomes of *Ciconiphilus* sp. ex [white-faced heron], *Colpocephalum* sp. 1 ex [Australian pelican], *Colpocephalum* sp. 2 ex [straw-necked ibis], *Eomenopon* sp. ex [scaly-breasted lorikeet], *Franciscoloa* sp. 1 ex [yellow-tailed black cockatoo], *Franciscoloa* sp. 2 ex [sulfur-crested cockatoo], *Franciscoloa* sp. 3 ex [little corella, galah and pheasant coucal], *Piagetiella* sp. ex [Australian pelican], *Plegadiphilus* sp. ex [Australian white ibis], *Laemobothrion* sp. 1 ex [Eurasian coot], *Laemobothrion* sp. 3 ex [Australian swamphen] and *Laemobothrion* sp. 2 ex [black kite]. Gene names are: *atp6* and *atp8* for ATP synthase subunits 6 and 8; *cob* for cytochrome b; *cox1-3* for cytochrome c oxidase subunits 1-3, *nad1-5* and *nad4L* for NADH dehydrogenase subunits 1-5 and 4L; *rrnS* and *rrnL* for small and large subunits of ribosomal RNA. tRNA genes are indicated with their single-letter abbreviations of the corresponding amino acids. Genes are transcribed from left to right except those underlined, which have an opposite orientation of transcription. Non-coding regions are in black. Circular mitochondrial genomes are linearised for the sake of illustration.

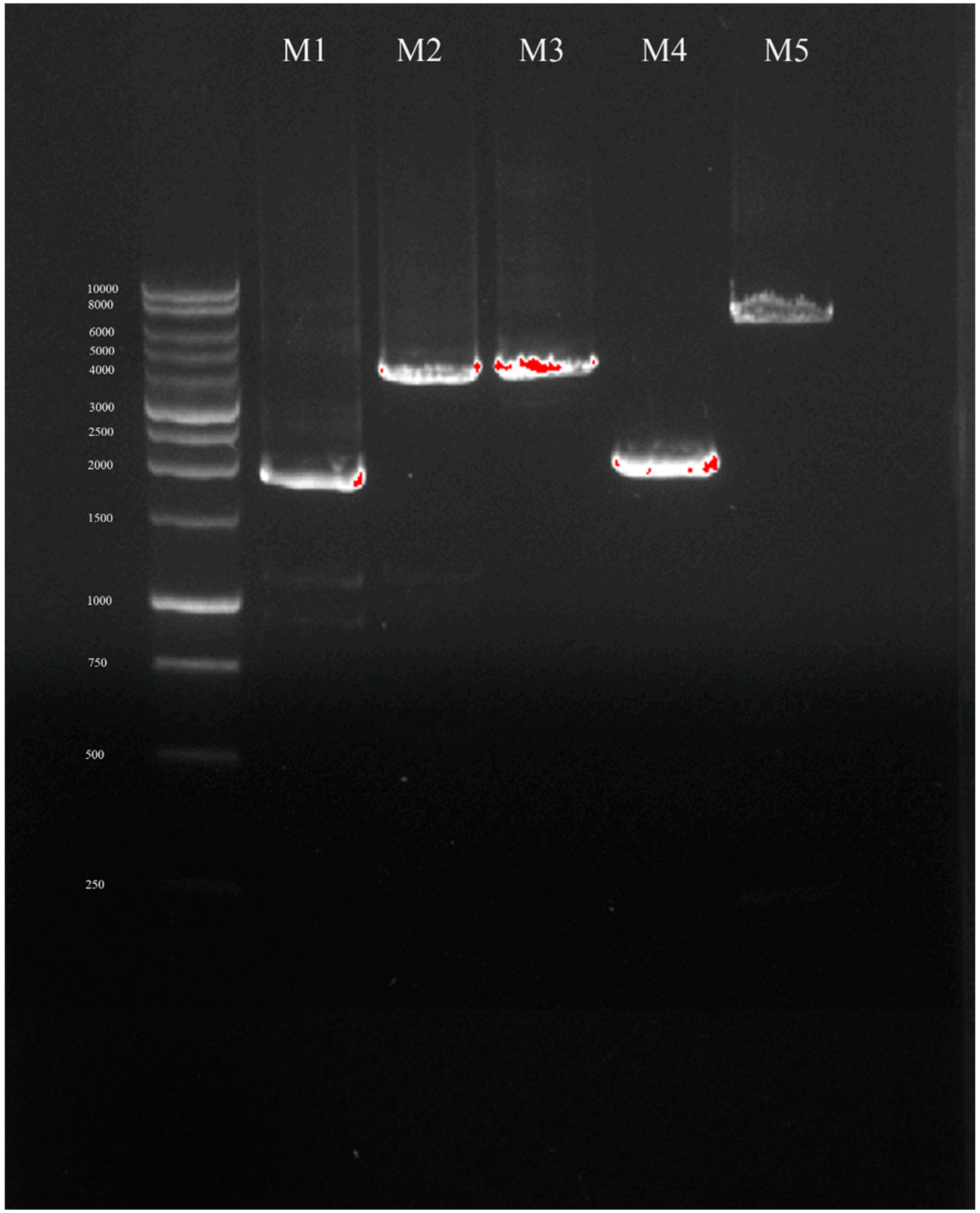

Supplementary Figure S2. PCR verification of the five mitochondrial minichromosomes (M1-M5) of *Actornithophilus* sp. 1 ex [pied oystercatcher]. Marker band sizes (bp) are indicated on the left side.

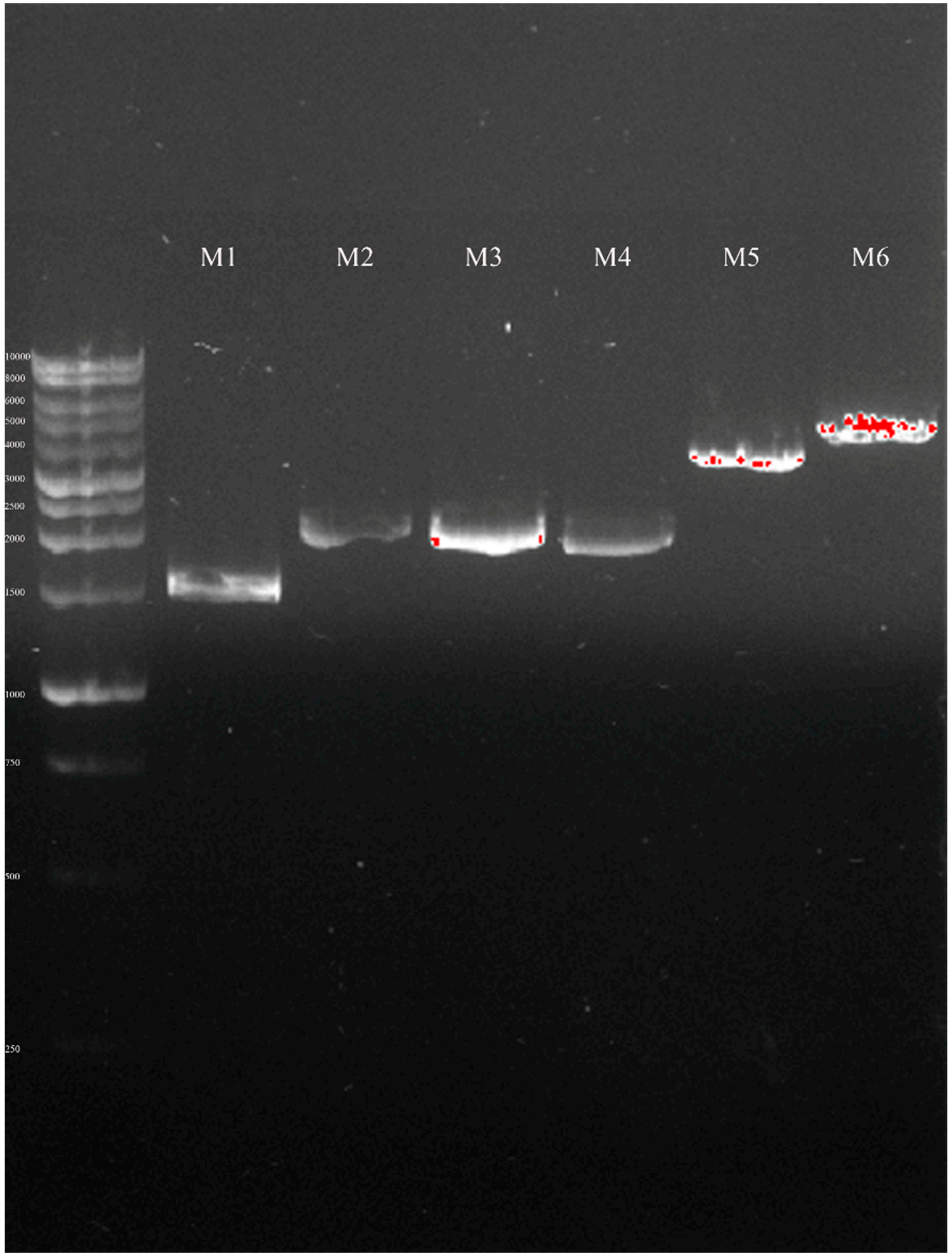

Supplementary Figure S3. PCR verification of the six mitochondrial minichromosomes (M1-M6) of *Actornithophilus* sp. 2 ex [masked lapwing]. Marker band sizes (bp) are indicated on the left side.

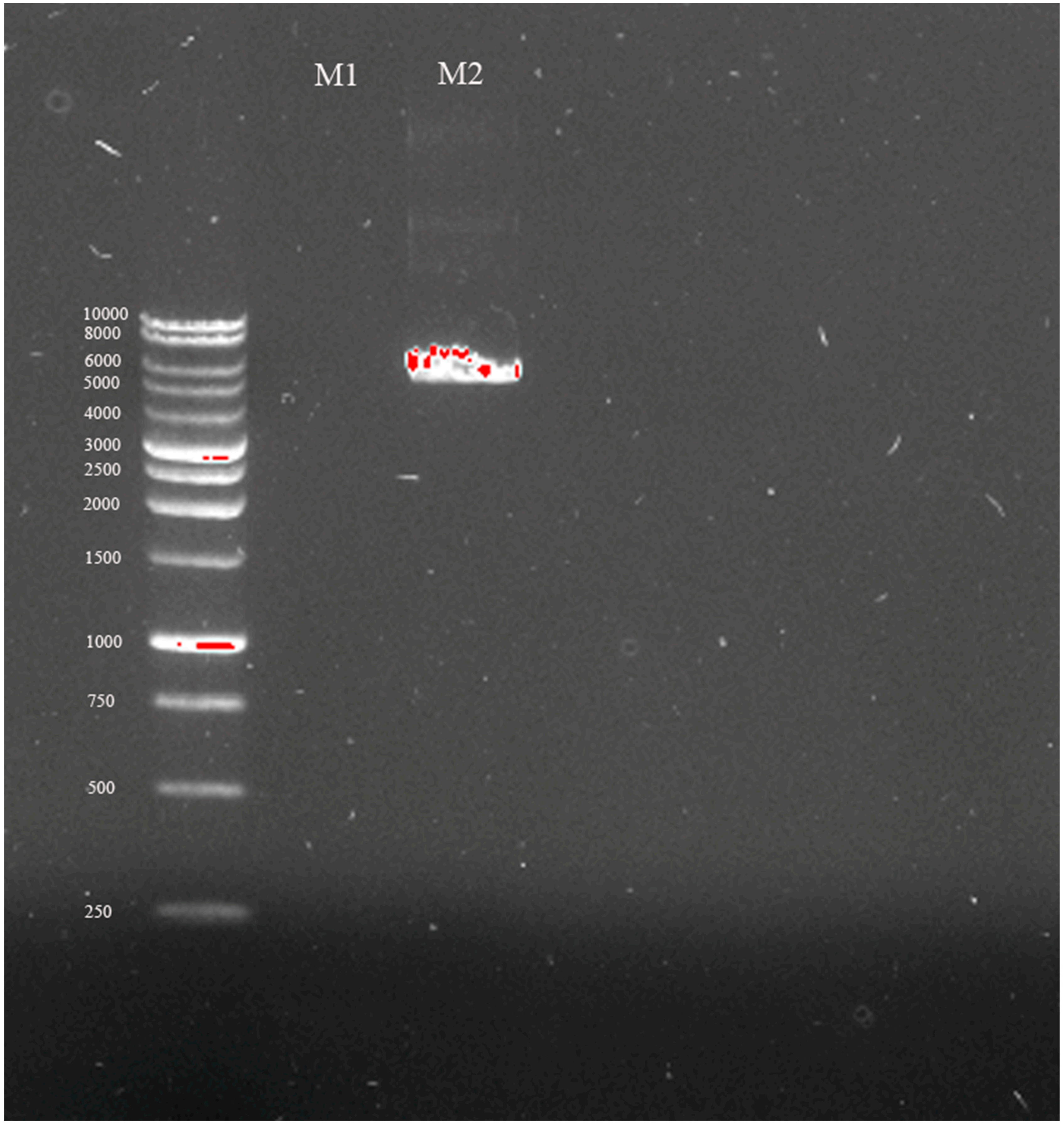

Supplementary Figure S4. PCR verification of the two mitochondrial minichromosomes (M1 and M2) of *Austromenopon* sp. 2 ex [sooty tern and crested tern]. Marker band sizes (bp) are indicated on the left side.

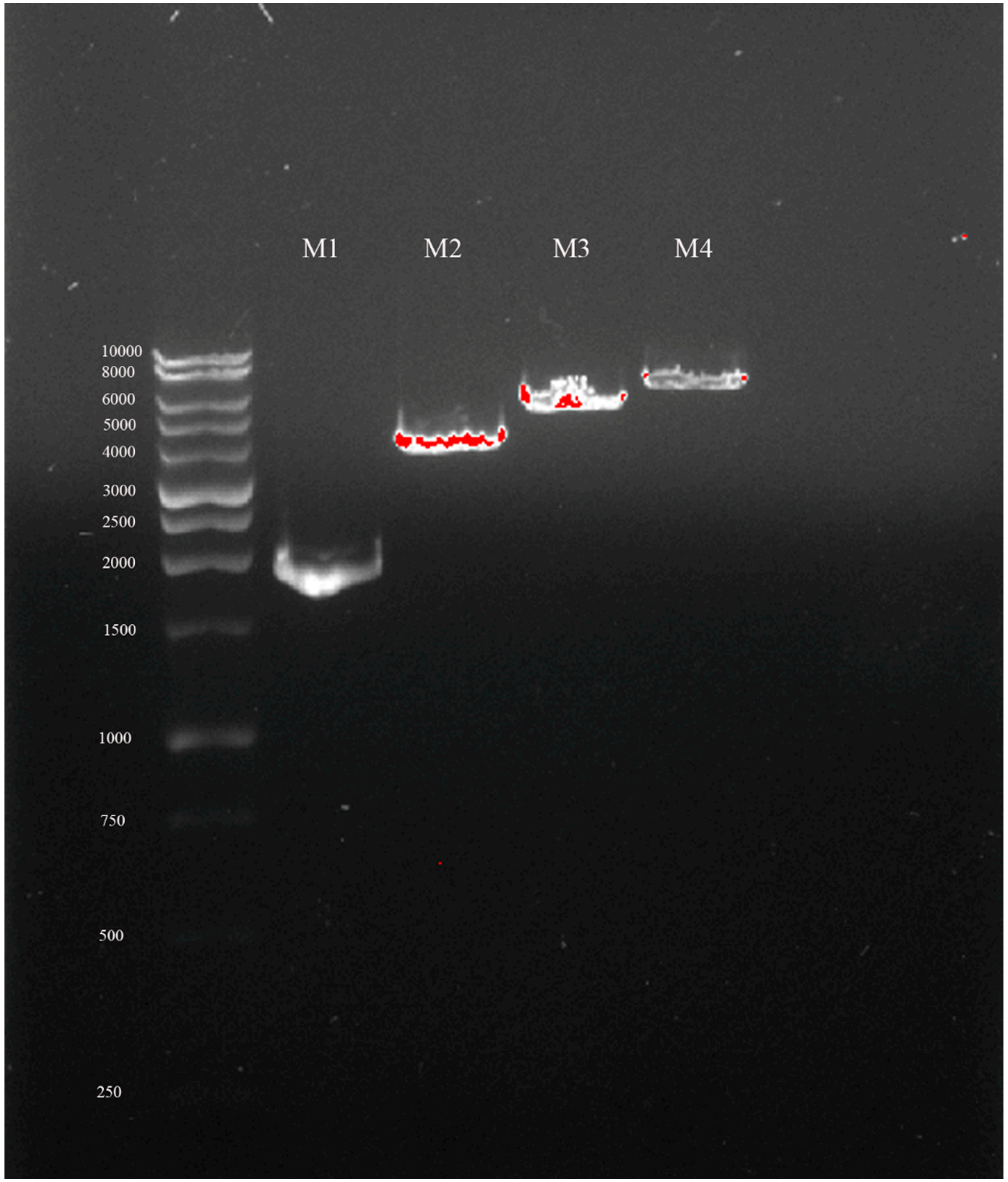

Supplementary Figure S5. PCR verification of the four mitochondrial minichromosomes (M1-M4) of *Myrsidea* sp. 1 ex [satin bowerbird]. Marker band sizes (bp) are indicated on the left side.

Supplementary Table S1. Specific primers used for PCR verification of mitochondrial minichromosomes and the number of Illumina sequence reads obtained from each PCR amplicon

Minichromosome	Species	Forward prime	Reverse Prime	Number of reads
E-nad4L-nad4	*Actornithophilus* sp. 1 ex [pied oystercatcher]	AAGGGTTAGGGAGTTTTTACTAGGAATAAG	GAAGTTAACCCATGAGAAATTATGAGGATC	19,380,282
F-S_1-nad2-L1-W-S2-H-nad6	*Actornithophilus* sp. 1 ex [pied oystercatcher]	CCGAGTGGAGGAAGGGAAGGAACCT	CAGAAAGGGTGGTGGAGCTCGAGAAG	16,833,026
V-K-cob-nad1-Q-A-nad3	*Actornithophilus* sp. 1 ex [pied oystercatcher]	GATGATTCAAAACCACATTCAAAAGGAGAA	GATGATTCAAAACCACATTCAAAAGGAGAA	18,067,258
rrnL-rrnS	*Actornithophilus* sp. 1 ex [pied oystercatcher]	CTTCCCTTGTTTAACCTTTCATTCCATCAC	CTGATGGTAGACGAGAAGACCCTATAGATC	16,228,942
Y-atp8-atp6-N-nad5-M-D-T-L_2-G-I-cox2-cox1-C-cox3	*Actornithophilus* sp. 1 ex [pied oystercatcher]	GTAGCAGGTTACATAATCCATTCTTCTTCC	ATCTACTTCTACTAACCCTCTTAGCCTAGC	20,100,600
E-nad4L-nad4	*Actornithophilus* sp. 2 ex [masked lapwing]	ATTTTTGATTACCTAAAGCCCACGTTGAGG	GAAGCTTCAATTCGTTCAGGTTGGTAAC	16,105,462
V-K-cob-nad1	*Actornithophilus* sp. 2 ex [masked lapwing]	ATCGAGTATGGGGGGATTATGTACACTATG	CAATTGATCGAACCCTTCCTAACATAGC	16,120,914
nad3-A-W	*Actornithophilus* sp. 2 ex [masked lapwing]	GGTATCCCTGTGGTGGTTGTATCTTTATAG	CCTAAAATGGTCTTACTCATACCCCTCAG	16,461,668
L_1-Q-rrnS-rrnL	*Actornithophilus* sp. 2 ex [masked lapwing]	CCTTAGCCCTTCCTCCTTTATTTAAACTTC	GGCAAACGCTTTTCCTGACTGTTTAGC	18,651,904
F-S_1-nad2-L_2-D-nad5-S_2-H-nad6	*Actornithophilus* sp. 2 ex [masked lapwing]	ATACAAACCTAACAACAACCATCTCCTATG	GATATTGGTTGCTACTCTATGGGTTAGG	17,282,038
Y-atp8-atp6-N-M-T-G-I-cox2-cox1-C-cox3	*Actornithophilus* sp. 2 ex [masked lapwing]	AATTCGAGTTGAGTTAGCTTTTCCAGGGAC	CCTAAAAGCCCAGACCAAATTCCAAATAAC	17,162,222
atp8-atp6-cox1-cox2-G-R-nad1-nad6-L_2-N-cob-cox3-H-I-nad5-nad2	*Austromenopon* sp. 2 ex [sooty tern and crested tern]	GTCCCTCACATTATAAGAGACTGAGTC	GAGCTTTTCTTGGACTCAGGTAAACTTTC	N.A.
K-Q-nad4L-nad4-E-T-V-A-C-W-nad3-Y-D-S_1-M-F-rrnL-rrnS-L_1-P	*Austromenopon* sp. 2 ex [sooty tern and crested tern]	TCGTACTTTGAGATATCTGGTTCTTTAGG	ATTAGCCTTTCTTTCCATGGACCATTATGC	18,149,704
rrnL	*Myrsidea* sp. 1 ex [satin bowerbird]	AGTTCCGCGGCTCTTTAAAACTTCAGTGAG	CATGGTAGACGAGAAGACCCTGTAGATC	16,316,178
rrnS	*Myrsidea* sp. 1 ex [satin bowerbird]	GCCCACCGCCGTCTATGTTAACTCAGGAG	GTGTATCTTCGTTTACTGAGCAGTTCCTC	18,548,112
Y-nad5-L_1-nad3-S_1-cox2-cox1-cox3	*Myrsidea* sp. 1 ex [satin bowerbird]	ATGGGAGCAATTAACTTTATCTCTACCCTC	GTAAAGAGAAAATAGCCATATCCACGGAAG	17,422,022
M-R-G-cob-Q-atp6-atp8-nad4L-nad4-nad6-nad1-V-nad2	*Myrsidea* sp. 1 ex [satin bowerbird]	CTCCTGTGAAGATTAATTTTGTTTTTTTCG	TGGCTGATCCCACCCAAAATATAGCAAGG	16,211,864

Supplementary Table S2. The mitochondrial minichromosomes of four bird louse species (family Menoponidae) identified by assembly of Illumina sequence reads (whole genomic DNA sequencing)

Louse species (bird host)	Minichromosome	Size (bp)	Number of sequence-reads	Mean coverage
Actornithophilus sp. 1 ex [pied oystercatcher]	*E-nad4L-nad4*	2,005	27,366	1,900
	V-K-cob-nad1-Q-A-nad3	3,950	77,869	2,956
	rrnL-rrnS	3,939	115,421	4,396
	nad6-H-S_2*-W-*L_1*-nad2-*S_1*-F*	2,068	23,907	1,720
	Y-atp8-atp6-N-nad5-M-D-T-L_2*-G-I-cox2-cox1-C-cox3*	6,112	21,714	2,023
Actornithophilus sp. 2 ex [masked lapwing]	*E-nad4L-nad4*	1,698	7,177	634
	V-K-cob-nad1	2,215	14,085	955
	A-W-nad3	2,102	19,763	1,406
	rrnL-rrnS-Q-L_1	2,125	43,077	3,036
	nad6-H-S_2*-nad5-D-*L_2*-nad2-*S_1*-F*	3,645	55,351	2,278
	Y-atp8-atp6-N-M-T-G-I-cox2-cox1-C-cox3	4,361	67,577	2,323
Austromenopon sp. 2 ex [sooty tern and crested tern]	*cox2-cox1-atp8-atp6-nad2-nad5-I-H-cox3-cob-N-*L_2*-nad6-nad1-R-G*	9,499	99,206	1,567
	nad4L-nad4-E-T-V-A-C-W-nad3-Y-D-S_1*-M-F-rrnL-rrnS-*L_1*-P-K-*S_1*-Q*	4,943	54,918	1,665
Myrsidea sp. 1 ex [satin bowerbird]	*rrnL*	1,969	5,551	422
	rrnS	3,194	41,381	1,595
	Y-nad5-L_1*-nad3-S1-cox2-cox1-cox3*	5,167	58,205	1,690
	M-R-G-cob-Q-atp8-atp6-nad4L-nad4-nad6-mad1-nad2	6,185	68,633	1,665

Supplementary Table S3. Derived minichromosomes and gene clusters observed in Menoponidae and Laemobothriidae species

Family	Species	Fragmented	*E-nad4L-nad4* (as one minichromosome)	*V-K-cob-nad1*	*nad6*-$\underline{H}$-$\underline{S_2}$	*nad2*-S_1-$\underline{F}$	*Y-atp8-atp6-N*	*G*-$\underline{I}$-*cox2-cox1*-$\underline{C}$-*cox3*	*cox2-cox1*-$\underline{atp6\text{-}atp8}$	*nad2-nad5-I*	*nad3-Y*	*rrnL-rrnS*-L_1
Menoponidae	*Actornithophilus* sp. 1 ex [pied oystercatcher]	+	+	+	+	+	+	+	-	-	-	-
	Actornithophilus sp. 2 ex [masked lapwing]	+	+	+	+	+	+	+	-	-	-	-
	Amyrsidea (Argimenopon) minuta (Emerson, 1961)	-	-	-	-	-	-	-	-	-	-	-
	Austromenopon sp. 2 ex [sooty tern and crested tern]	+	-	-	-	-	-	-	+	+	+	+
	Austromenopon sp. 1 ex [sooty shearwater]	-	-	-	-	-	-	-	+	+	+	+
	Ciconiphilus sp. ex [White-faced heron]	-	-	-	-	-	-	-	-	-	-	-
	Colpocephalum sp. 1 ex [Australian pelican]	-	-	-	-	-	-	-	-	-	-	-
	Colpocephalum griffoneae (Ansari, 1955)	-	-	-	-	-	-	-	-	-	-	-
	Colpocephalum sp. 2 ex [straw-necked ibis]	-	-	-	-	-	-	-	-	-	-	-
	Eomenopon sp. ex [scaly-breasted lorikeet]	-	-	-	-	-	-	-	-	-	-	-
	Franciscoloa sp. 1 ex [yellow-tailed black cockatoo]	-	-	-	-	-	-	-	-	-	-	-
	Franciscoloa sp. 2 ex [sulfur-crested cockatoo]	-	-	-	-	-	-	-	-	-	-	-
	Franciscoloa sp. 3 ex [little corella, galah and pheasant coucal]	-	-	-	-	-	-	-	-	-	-	-
	Menacanthus cornutus (Schömmer, 1913)	-	-	-	-	-	-	-	-	-	-	-
	Myrsidea sp. 1 ex [satin bowerbird]	+	-	-	-	-	-	-	-	-	-	-
	Myrsidea sp. 2 ex [citrine warbler]	+	-	-	-	-	-	-	-	-	-	-
	Osborniella crotophagae (Stafford, 1943)	-	-	-	-	-	-	-	-	-	-	-
	Piagetiella sp. ex [Australian pelican]	-	-	-	-	-	-	-	-	-	-	-
	Plegadiphilus sp. ex [Australian white ibis]	-	-	-	-	-	-	-	-	-	-	-
Laemobothriidae	*Laemobothrion* sp. 1 ex [Eurasian coot]	-	-	-	-	-	-	-	-	-	-	-
	Laemobothrion sp. 2 ex [black kite]	-	-	-	-	-	-	-	-	-	-	-
	Laemobothrion sp. 3 ex [Australian swamphen]	-	-	-	-	-	-	-	-	-	-	-
	Laemobothrion (Laemobothrion) tinnunculi (Linnaeus, 1758)	+	-	-	-	-	-	-	-	-	-	-

Supplementary Table S3 (continued)

Family	Species	*nad4L-nad4-E*	*P-K-S*$_1$	*Y-nad5-L1-nad3-S*$_1$*-cox2-cox1-cox3* (as one minichromosome)	*G-cob-Q-atp6-atp8-nad4L-nad4-nad6-nad1*	*D-rrnL-S*$_2$	*E-rrnS-L*$_2$	*nad5-nad6-C-nad4L-nad4-Y-atp8-atp6*
Menoponidae	*Actornithophilus* sp. 1 ex [pied oystercatcher]	-	-	-	-	-	-	-
	Actornithophilus sp. 2 ex [masked lapwing]	-	-	-	-	-	-	-
	Amyrsidea (Argimenopon) minuta (Emerson, 1961)	-	-	-	-	-	-	-
	Austromenopon sp. 2 ex [sooty tern and crested tern]	+	+	-	-	-	-	-
	Austromenopon sp. 1 ex [sooty shearwater]	+	+	-	-	-	-	-
	Ciconiphilus sp. ex [White-faced heron]	-	-	-	-	-	-	-
	Colpocephalum sp. 1 ex [Australian pelican]	-	-	-	-	-	-	-
	Colpocephalum griffoneae (Ansari, 1955)	-	-	-	-	-	-	-
	Colpocephalum sp. 2 ex [straw-necked ibis]	-	-	-	-	-	-	-
	Eomenopon sp. ex [scaly-breasted lorikeet]	-	-	-	-	-	-	-
	Franciscoloa sp. 1 ex [yellow-tailed black cockatoo]	-	-	-	-	-	-	-
	Franciscoloa sp. 2 ex [sulfur-crested cockatoo]	-	-	-	-	-	-	-
	Franciscoloa sp. 3 ex [little corella, galah and pheasant coucal]	-	-	-	-	-	-	-
	Menacanthus cornutus (Schömmer, 1913)	-	-	-	-	-	-	-
	Myrsidea sp. 1 ex [satin bowerbird]	-	-	+	+	-	-	-
	Myrsidea sp. 2 ex [citrine warbler]	-	-	+	+	-	-	-
	Osborniella crotophagae (Stafford, 1943)	-	-	-	-	-	-	-
	Piagetiella sp. ex [Australian pelican]	-	-	-	-	-	-	-
	Plegadiphilus sp. ex [Australian white ibis]	-	-	-	-	-	-	-
Laemobothriidae	*Laemobothrion* sp. 1 ex [Eurasian coot]	-	-	-	-	+	+	+
	Laemobothrion sp. 2 ex [black kite]	-	-	-	-	+	+	+
	Laemobothrion sp. 3 ex [Australian swamphen]	-	-	-	-	+	+	+
	Laemobothrion (Laemobothrion) tinnunculi (Linnaeus, 1758)	-	-	-	-	+	+	+
